# Tailored Iron Oxide Nanoparticles as Potential Cannabinoid Carriers for Anti-Cancer Treatment

**DOI:** 10.3390/biom15020230

**Published:** 2025-02-05

**Authors:** Jan Taudul, Joanna Celej, Kinga Żelechowska-Matysiak, Daria Kępińska, Agnieszka Majkowska-Pilip, Marcin Strawski, Paweł Krysiński, Dorota Nieciecka

**Affiliations:** 1 Faculty of Chemistry, University of Warsaw, Ludwika Pasteura 1, 02-093 Warsaw, Poland; j.taudul@uw.edu.pl (J.T.); marcin@chem.uw.edu.pl (M.S.); 2 Institute of Physical Chemistry of the Polish Academy of Sciences, Marcina Kasprzaka 44/52, 01-224 Warsaw, Poland; jcelej@ichf.edu.pl; 3 Centre of Radiochemistry and Nuclear Chemistry, Institute of Nuclear Chemistry and Technology, Dorodna 16, 03-195 Warsaw, Poland; k.zelechowska@ichtj.waw.pl (K.Ż.-M.); a.majkowska@ichtj.waw.pl (A.M.-P.); 4 Biotech Evolution, Mokra 3C, 05-092 Łomianki, Poland; d.kepinska@hempevolution.eu

**Keywords:** iron oxide nanoparticles, nanocarriers, cannabinoids, epirubicin

## Abstract

We present a novel, multicomponent nanoparticulate carrier system based on superparamagnetic iron oxide nanoparticles with a designed hydrophilic/hydrophobic balance based on oleic acid and TWEEN 80 to incorporate hydrophobic cannabinoids—cannabigerol and cannabidiol—as well as the hydrophilic anthracycline drug epirubicin, forming a conjugate anticancer system. Additionally, the superparamagnetic iron oxide-based nanoparticles formed the core of the system, thus providing it with magnetic hyperthermia capabilities with a specific absorption rate comparable to the corresponding systems in the literature. The interaction of the conjugate with the cell membrane was studied using the Langmuir monolayers at the air/water interface formed of selected lipids modeling the healthy and cancerous cell membranes. Finally, cytotoxicity tests were carried out against the SKOV-3 cell line in vitro. A synergistic effect was observed when both the cannabinoid and epirubicin were present in the conjugate, as compared to the cannabinoid or epirubicin alone, making our system advantageous for further development for tentative therapeutic use.

## 1. Introduction

Superparamagnetic iron oxide-based nanoparticles (SPIONs) are one of the most popular and well-examined types of nanoparticles. They can show behavior different from typical crystals or atoms [1] like notable stability, solubility, and higher chemical or biological activity. They can be radically altered by coating them with various substances, and thanks to that we can create structures with previously unseen properties [2]. These nanoparticles demonstrate many new features such as huge specific surface area, increased chemical [3] and mechanical resistance [4,5], adsorption and absorption capacity [6], and hydrophilicity [7]. Cutting-edge properties of SPIONs, such as biocompatibility, anti-inflammatory and antibacterial activity, effective drug delivery, bioactivity, bioavailability, tumor targeting, and bio-absorption, have led to a growth in the biotechnological, and applied microbiological applications of SPIONs [8]. Their magnetic properties have been found to be useful in targeted therapy [9,10,11]. Using the external magnetic field, we can control SPIONs to a specific place in the patient’s body. Additionally, the superparamagnetic core can be used therapeutically, creating localized magnetic hyperthermia under the influence of an external, alternating magnetic field, SPIONs will selectively heat the tumor tissue, inducing the death of the cancer cells [12]. Because of the nanoparticles’ properties, the delivery is more efficient and the drug dose is reduced, which minimizes side effects [13].

An extensive research topic of recent years are also cannabinoids. The medicinal properties of the *Cannabis sativa* L. plant have been known to humankind for hundreds of years. Still, only in the last 20 years, the research on the medicinal properties of cannabinoids has progressed and important discoveries have been made. The main one was the discovery of the cannabinoid receptors CB_1_ and CB_2_ in the human body. They share 48% of the amino acid sequence, but they differ in the signalization mechanism and the distribution in tissues or sensitivity to some antagonists that are selective for one of the receptors [14]. CB_1_ receptors are located in the central nervous system, on the surface of liver adipocytes, pancreas, and skeletal muscles [14]. CB_2_ receptors are expressed on the surface of cells belonging to the immune system, mainly lymphocytes B, NK cells, lymphocytes T, monocytes, macrophages, and microglia cells. Receptors can also be found on the surface of cancer cells (so-called overexpression of these receptors) [15]. Because of the psychoactive properties of some of the cannabinoids, in medicine, we use inactive isomers [16]. Two of the inactive isomers are cannabidiol (CBD) and cannabigerol (CBG). Both of these isomers have similar properties. CBD is administered to patients suffering from seizures [17], anxiety [18], addictions [19], or chronic illnesses [20], while CBG additionally helps with the patient’s eyesight [21]. Cannabinoids have anti-cancer properties. They are also used to heal cancer pain [22] which is why they are being paired with different drugs like anthracyclines [23], with which they might show a synergistic effect [24]. It seems that the cytotoxic effect of cannabinoids may be due among other things, such as their influence on the functioning of ion channels, including the calcium channel. Dysregulation of calcium homeostasis causes mitochondrial Ca^2+^ overload and disturbances in the properties of the mitochondrial membrane, which may result in increased reactive oxygen species (ROS) production. The aforementioned overproduction of ROS promotes reactions with cell components, including phospholipids of membranes containing unsaturated fatty acids. It causes changes in the structure of cell membranes and, consequently, in their physicochemical properties, which may lead to changes in the functioning of biomembranes. However, due to hydrophobic properties cannabinoids, the usage of them for medical purposes can be limited. Therefore, there exists a pressing need to develop a platform capable of a safe and appropriate delivery system overcoming the hydrophobicity barrier of cannabinoids.

For that reason, the goal of this project was to synthesize a nanoparticulate carrier system of carefully designed hydrophilic/hydrophobic balance based on SPIONs. The size of the core of obtained nanostructures was examined using Transmission Electron Microscopy, whereas the dynamic light scattering technique and the Zeta potential measurement provided information about their hydrodynamic size and stability in an aqueous environment. Infrared spectroscopy, cyclic voltammetry, and differential scanning calorimetry were used to confirm the attachment of the cannabinoid and epirubicin to the carrier. Cyclic voltammetry and differential scanning calorimetry were also utilized to quantify the amount of the attached cannabinoid and drug in the conjugate. Next, the interactions between the released drug and model biological membranes were studied with the Langmuir trough. Additionally, the heat induction ability of the obtained carriers was tested using magnetic hyperthermia. The final stage of the project was to determine the cytotoxicity of nanocarriers using the MTS test on the SK-OV-3 ovarian cancer cell line.

## 2. Materials and Methods

### 2.1. Chemicals

Iron (III) chloride hexahydrate FeCl_3_·6H_2_O Aldrich ACS reagent 97%, and iron (II) chloride tetrahydrate FeCl_2_·4H_2_O puriss p.a. ≥ 99% (RT), were supplied from Sigma-Aldrich (Sigma-Aldrich, St. Louis, MO, USA), and 25% ammonia solution NH_4_OH and chloroform were supplied from POCH (POCH, Wrocław, Poland). Deionized water with a resistivity of 18.2 MΩ cm at 25 °C was obtained using the Milli-Q ultra-pure water filtering system from Merck (Merck, Warszawa, Poland). Oleic acid technical grade 90% and TWEEN 80 were supplied by Sigma-Aldrich (Sigma-Aldrich, St. Louis, MO, USA). Lipids DOPC (1,2-dioleoyl-sn-glycero-3-phosphocholine) and DOPS (1,2-dioleoyl-sn-glycero-3-phosphoserine) were purchased from Avanti Polar. CBD and CBG isolates were obtained as a gift from Biotech Evolution. The epirubicin hydrochloride European Pharmacopoeia (EP) Reference Standard was purchased from Sigma-Aldrich.

### 2.2. Synthesis of the Conjugates

#### 2.2.1. Synthesis of SPIONs

The synthesis of SPIONs was performed with the coprecipitation technique [25]. Briefly, Fe^3+^ and Fe^2+^ salts were dissolved in 50 and 40 mL of distilled water, respectively. Dissolution was assisted with a magnetic stirrer. Ammonia was added dropwise to the combined solutions. The orange solution began to turn black due to the black precipitate of iron oxide nanoparticles. Ammonia was added until the pH was approximately 10 [26]. Using the magnetic properties of nanoparticles, the beaker with the obtained solution was placed on a neodymium magnet to separate phases. The supernatant was decanted from above the nanoparticle sediment. The precipitate was washed several times with water to remove any undissolved iron salts or ammonia residues.

#### 2.2.2. Modification of SPIONs with Oleic Acid

To attach TWEEN 80 and the cannabinoid to the structure, the surface of the nanoparticles must be changed from hydrophilic to hydrophobic. The previously obtained nanoparticles were suspended in 20 mL of water and sonicated for 15 min in a 0.5 s cycle. At the same time, the hotplate was heated to 80 degrees. The solution was then placed on a plate and mechanically mixed. Then 2 milliliters of oleic acid were added. Oleic acid is used to modify the surface of nanoparticles to make them hydrophobic, but it will also make the nanoparticles less stable. Due to their hydrophobicity, nanoparticles will begin to precipitate. The beaker was heated with stirring for one hour.

#### 2.2.3. Modification of SPIONs with TWEEN 80 and Cannabinoid

Meanwhile, 200 mg of cannabinoid was mixed with 3.5 mL of TWEEN 80. TWEEN 80 not only changes the surface’s nature but also locks cannabinoid molecules between the chains of oleic acid and the hydrophobic part of the surfactant. TWEEN 80 also improves crossing the blood–brain barrier [27]. Previously prepared TWEEN 80 with cannabinoid was added to the reaction mixture and stirring was continued for another hour [28]. The TWEEN 80 will attach to the oleic acid chains creating a lipid bilayer, in which the cannabinoid is locked.

#### 2.2.4. Modification of the Conjugate with Epirubicin

The last stage of the synthesis was the incorporation of epirubicin into the conjugate. Firstly, 4 mg of epirubicin was dissolved in 200 μL of distilled water. Then, 600 μL of the conjugate was added to the epirubicin solution, mixed, and left for 30 min to adsorb the anthracycline on/in the layer surrounding SPIONs.

### 2.3. In Vitro Cytotoxicity Evaluation

Cytotoxicity studies were conducted on nanoparticle dispersions containing: SPION@OA_T80, conjugated with CBD (SPION@OA_T80_CBD), or CBG (SPION@OA_T80_CBG), and their combination with epirubicin (SPION@OA_T80_CBD_epi, and SPION@OA_T80_CBG_epi). Pure epirubicin was also tested. Nanoparticle concentrations ranged from 0.5 to 4 µg/mL corresponding to 0.05–0.4 µg/mL of cannabinoids and Epi (10% *w*/*w*). SKOV-3 cells were purchased from the American Type Tissue Culture Collection (ATCC, Rockville, MD, USA) and cultured in McCoy’s 5A medium supplemented with 10% FBS and 1% penicillin–streptomycin (all from Beth Haemek, Israel). SKOV-3 cells were seeded into 96-well plates at a density of 2.5 × 10^3^ cells per well and incubated at 37 °C in a humidified environment with 5% CO_2_. After 24 h, the cells were washed with PBS, and various concentrations of SPIONs/CBD/CBG/epi compounds were added. The treated cells were then incubated for an additional 24, 48, and 72 h. Cytotoxicity was assessed using the MTS assay with the CellTiter-96^®^ AQueous One Solution Cell Proliferation Assay kit (Promega Corporation, Madison, WI, USA). The absorbance of the formazan product was measured at 490 nm using a microplate reader (Berthold Technologies, Bad Wildbad, Germany). Results are expressed as the percentage of viable cells relative to untreated control cells.

### 2.4. Langmuir Technique

After thoroughly cleaning the Langmuir trough with ethanol and chloroform, it was filled with MilliQ water. Then, the chloroform lipid solution was dropped onto the surface of the subphase and 10 min were left for the organic solvent to evaporate. After this time, the compression of the monolayer using barriers was started until the surface pressure of 15 or 30 mN/m was reached. After reaching this value, the barriers were stopped, the conjugate was injected into the subphase and the changes in surface pressure were recorded for approx. 4 h. The injected amounts of conjugate solutions were to provide a final subphase concentration of 8 µg/mL. These measurements provide information on the effect of the conjugates and their components on the stability of the monolayer in time.

### 2.5. Techniques

The morphology of SPIONs was investigated by Dynamic Light Scattering (DLS) for analyzing the hydrodynamic size of SPIONs and hybrids. TEM measurements (Libra 120 microscope, Zeiss, Oberkochen, Germany) were also used to assess the size of the conjugates. Before the measurement, a drop of the suspension was placed on a formvar-coated copper grid and allowed to dry in the air.

The modification of the SPIONs’ surface was characterized by FTIR spectroscopy with Nicolet 8700 Spectrometer Fisher Scientific. The analysis with Differential Scanning Calorimetry was performed with DSCQ20. The material was also investigated by X-ray photoelectron spectroscopy (XPS) recorded on a Kratos Axis Supra spectrometer (Kratos Analytical Ltd., Manchester, UK), equipped with a monochromatic Al Kα radiation source (1486.7 eV). All survey data were collected with an analyzer with a pass energy of 80 eV. The value of 20 eV was used for high-resolution spectra, except nitrogen region where the value of 40 eV was chosen. The effect of sample charging was reduced by a co-axial neutralization system. The occurring shift of the energy scale was corrected by setting the main component of C1s at the literature value of 284.8 eV for adventitious carbon [29]. Peak fitting of the data was performed with the use of a Shirley background type. A convolution of GL(30) line shapes was used to fit the individual peaks. Electrochemical experiments were performed using a commercial Workstation (CH Instruments C660, Austin, TX, USA) with a small-volume three-electrode cell with a glassy carbon electrode (GCE, 3 mm diameter, BASi MF-2012, West Lafayette, IN, USA) and a Pt mesh counter electrode. All potentials are reported relative to the Ag,AgCl|1 M KCl_aq_ reference electrode. The Magnetic hyperthermia (MH) measurements were performed with nanoScaleBiomagnetics D5 Series equipment with CAL1 CoilSet. The Specific Absorption Rate (SAR) values were estimated using MaNIaC Controller 1.1 software with ZaR subprogram (nBnanoScaleBiomagnetics, Zaragoza, Spain). Langmuir monolayers were prepared with a KSV-Nima KN2003 trough system with the KSV NIMA LB software 3.7 (Biolin Scientific, Manchester, UK).

### 2.6. Statistical Analysis

Chou–Talalay interaction analysis was performed using CompuSyn 1.0 software [30]. CompuSyn software fits a least-squares cell viability curve from the experimental data. This procedure also allows for the evaluation of a Combination Index (CI). CI tells us about the type of interaction between two drugs. It was determined in relation to the therapeutic effect of the combination of cannabinoid and epirubicin, i.e., the degree of inhibition of cell growth. If the value of the CI is closer to 0, the interaction is synergistic. The higher the factor, the interaction becomes more antagonistic. CompuSyn software calculates CI using the experimental data, with the following Formula (1):

(1) $$CI=\frac{{\left(D\right)}_{1}}{{\left(Dx\right)}_{1}}+\frac{{\left(D\right)}_{2}}{{\left(Dx\right)}_{2}}$$

where the CI is the combination index; (*Dx*)_1_ and (*Dx*)_2_ are concentrations of substances 1 and 2 administered separately causing a specific therapeutic effect *x*, e.g., 50%; (*D*)_1_ and (*D*)_2_ are concentrations of substances 1 and 2 administered in a combination causing the same therapeutic effect as when administered separately.

The dose reduction index (DRI) was also calculated, which determines whether and by how much it is possible to reduce the concentration of the tested substance administered in combination to achieve the same therapeutic effect:

(2) $${\left(DRI\right)}_{1}=\frac{{\left(Dx\right)}_{1}}{{\left(D\right)}_{1}}\;{\left(DRI\right)}_{2}=\frac{{\left(D\right)}_{2}}{{\left(Dx\right)}_{2}}$$

where (DRI)_1_ and (DRI)_2_ are parameters for substances 1 and 2, respectively; (*Dx*)_1_ and (*Dx*)_2_ are concentrations of substances 1 and 2 administered separately, causing a specific therapeutic effect *x*, e.g., 50%; (*D*)_1_ and (*D*)_2_ are concentrations of substances 1 and 2 administered in combination causing the same therapeutic effect as when administered separately.

Statistical analysis of the cytotoxicity study data was performed using GraphPad Prism software, version 8.4.3 (GraphPad Software Inc., San Diego, CA, USA). A one-way ANOVA test was used for the statistical evaluation, with results expressed as means ± standard deviation. All samples were tested at least in triplicate. Statistical significance was assessed based on *p*-values, with the following thresholds: *p* ≤ 0.05 (*), *p* ≤ 0.01 (**), *p* ≤ 0.001 (***), and *p* ≤ 0.0001 (****).

## 3. Results and Discussion

### 3.1. Morphology Studies

**Figure 1:**
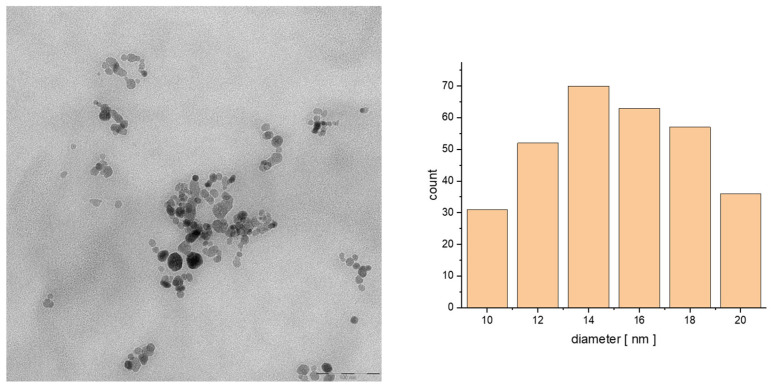
TEM images of the particle size distribution of SPION@OA_T80_CBD_epi (100 nm) and histogram of the size of the obtained structure.

**Figure 2:**
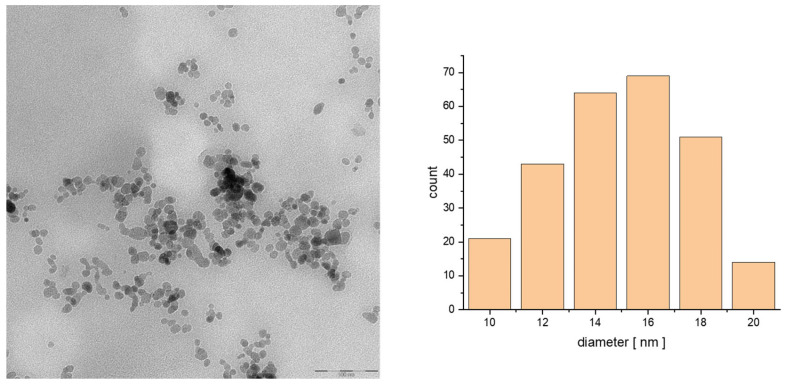
TEM images of the particle size distribution of SPION@OA_T80_CBG_epi (100 nm) and histogram of the size of the obtained structures.

The studies of morphology were performed using transmission electron microscopy. The hybrid material (prepared with a coprecipitation technique, consisting of spherical nanoparticles that have a size ranging from 10 to 20 nm as seen on prepared histograms) has a uniform surface and apparent aggregation. The aggregation presented on the TEM images is caused by the drying process onto a Formvar film covering the mesh for TEM analysis. As can be seen in Figure 1 and Figure 2, the SPIONs have quite regular morphology and dispersity similar to the following samples. We can see the organic shell covering the cores of the nanoparticles. We can conclude that the organic bilayer is capable of trapping a few small nanoparticles inside.

The Dynamic Light Scattering (DLS) technique was used to study the size of the prepared materials. The results of TWEEN 80-coated nanoparticles showed an average hydrodynamic diameter of about 65 (±3) nm. Modification of the nanoparticles with cannabinoids shows the growth of the diameter. For CBD the average diameter is 90 (±2) nm and for CBG 87 (±4) nm. Despite the different cannabinoids, the sizes are very similar. Because the cannabigerol molecule Is somehow smaller than the cannabidiol molecule, the carrier with CBG is smaller but it is within the margin of error. The diameter after epirubicin adsorption has increased. The CBD conjugate’s diameter totals 98 (±5) nm and for CBG’s conjugate 102 (±3) nm. The results of DLS measurements are included in Table 1.

The Zeta potential was measured at each step of SPIONs’ functionalization. The TWEEN 80-modified SPIONs have an average Zeta potential of 0.7 (±0.2) mV. TWEEN 80 is a molecule, which does not contain functional groups that will hydrolyze in an aqueous environment. Values of Zeta potential for CBD and CBG-modified nanoparticles are −12 (±4) and −13 (±5) mV, respectively. The addition of epirubicin causes the Zeta potential to increase to −2.7 (±1.2) mV for CBD and −3.9 (±1) mV for CBG. So, the overall changes are relatively small. The results of Zeta potential measurements are included in Table 1.

For medical applications of our conjugates, their stability in aqueous media as the suspension is of utmost importance. The stability of the nanoparticles was monitored by size analysis (DLS technique) and Zeta potential. The samples were tested for a period of 3 months. During this time, no significant deviations from the values presented in the Table 1 were observed, suggesting the lack of aggregation and precipitation of nanoparticles from the solution. However, based on the Zeta potential values, it is clear that hydrophilic interactions, not the surface charge, are responsible for its stability.

Additionally, the lack of aggregation of the conjugates and good stability of the suspension are also confirmed by the tested magnetic properties and low magnetic remanence value, which is discussed in more detail in the Supplementary Materials.

### 3.2. XPS Analysis

XPS technique was used to verify the formation of individual systems. Samples coated with an oleic acid layer, OA_T80 bilayer, and modified additionally with CBD and epirubicin were analyzed. Signals from the expected elements dominate the spectra, although some samples contain traces of elements that may come from process impurities or represent the substrate on which the samples were deposited [31]. Table 2 summarizes the average values for the respective samples. The oxygen and carbon ratio determined for the SPION@OA system is close to the ratio of 1:9, which coincides with the expected value for this system. We can conclude that the SPION system is so tightly covered by acid molecules that the signal from the magnetic core is very weak. Adding another layer (TWEEN 80) slightly disturbs our system. We observe the increase of a signal that can be assigned to iron. However, it is not significant, reaching a value of a single percent. The change in the oxygen-to-carbon ratio is more significant. This is consistent with the fact that TWEEN 80 is a compound with a significantly different O:C ratio of ca. 1:2.5. An increase in the oxygen amount value confirms the incorporation of this compound into the carrier structure. Subsequent modifications of the conjugate again change this ratio, this time in favor of carbon. The following incorporated compounds (CBD and epirubicin) have a higher carbon-to-oxygen ratio, which implies an increase in the value of the first element and a decrease in the second.

**Figure 3:**
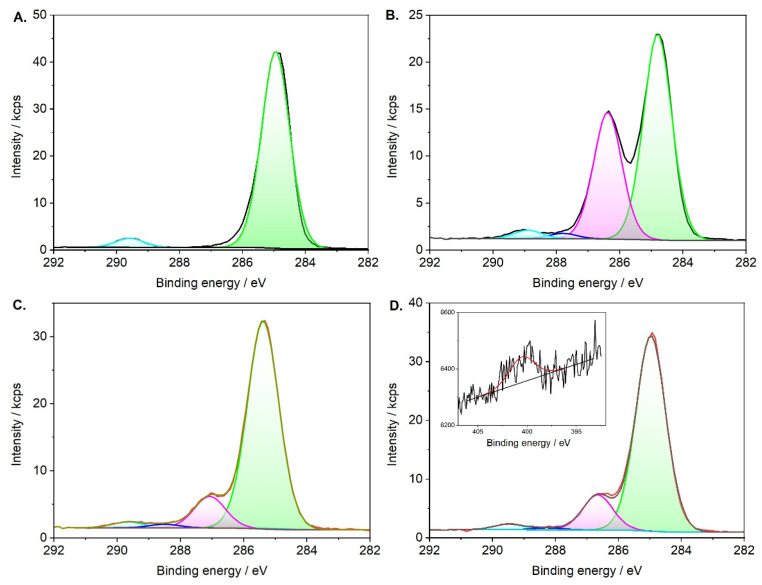
High-resolution C 1s XPS spectrum of (**A**) SPION@CA, (**B**) SPION@OA_T80, (**C**) SPION@OA_T80_CBD and (**D**) SPION@OA_T80_CBD_epi. The proposed model includes aliphatic carbon (green component), hydroxyl and/or ether-type C-O bond (magenta), carbonyl (blue), and carboxyl (light blue) carbon.

The above-described dependencies are also visible in the changes in high-resolution spectra. Figure 3 presents changes in the C 1s region for a series of samples. The structure for SPION@OA indicates a predominant amount of aliphatic carbon and a slight representation of the significantly oxidized form, the carboxyl group [32,33]. In the SPION@OA_T80 sample, a significant increase in the amount of carbon with an intermediate oxidation state—ether groups, which are the basic component of the T80 compound, is visible [33,34]. The last two systems are characterized by a decrease in the amount of these groups in relation to the other components. However, their representation is still present in each sample. This confirms the incorporation of subsequent molecules with a lower oxygen-to-carbon ratio. It is not possible to precisely distinguish between systems with CBD and EPI addition, because we are not able to predict their quantitative relationship. It can be admitted that uncertainty regarding epirubicin incorporation exists. Nevertheless, confirmation of its presence can be found in the high-resolution spectrum of the nitrogen region, for which in the case of the SPION@OA_T80_CBD_epi sample we can identify the appearance of the N 1s peak (Figure 3D insert). The low signal intensity is due to the simple relationship between the amount of nitrogen in the compound molecule and the remaining components, which are rich in oxygen and carbon.

### 3.3. FT-IR Studies

**Figure 4:**
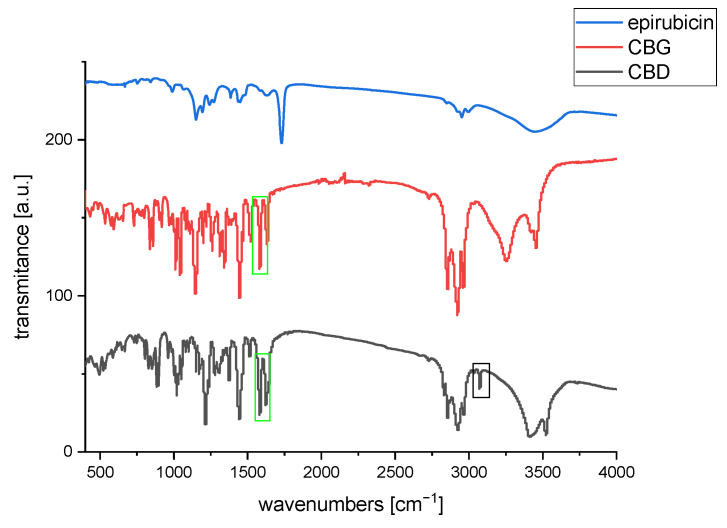
FT-IR spectrum for pure cannabinoids and epirubicin.

Fourier Transformed Infrared Spectroscopy was used to qualitatively identify functional groups of organic molecules on the conjugate’s surface and to confirm the presence of the iron oxide nanoparticles. For reference purposes, the spectra of pure cannabinoids (CBD, CBG), and epirubicin are presented in Figure 4.

**Figure 5:**
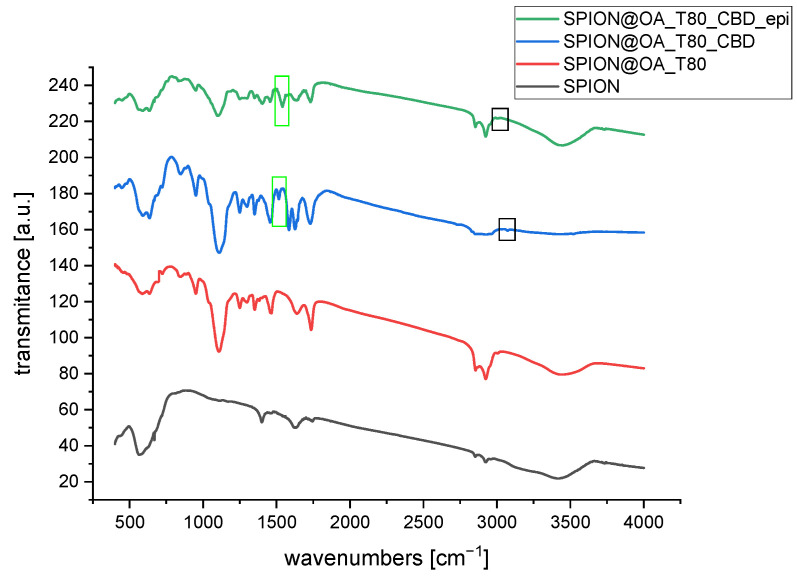
FT-IR spectra of obtained conjugates with CBD. In the green frame, the band coming from the C=C stretching of the phenyl ring; in the black frame, the vibration coming from the aromatic ring’s C-H bond are marked.

**Figure 6:**
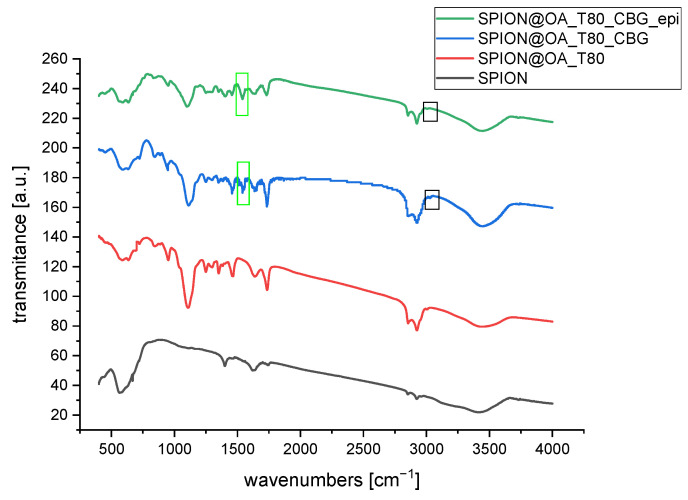
FT-IR spectra of obtained conjugates with CBG. Characteristic bands coming from the cannabinoids are marked. In the green frame: C=C stretching of the phenyl ring.

Figure 5 presents spectra for conjugates with CBD, while Figure 6 presents spectra for conjugates with CBG. As can be seen in the spectrum for bare nanoparticles, there appears a strong band in the range of 500 to 650 cm^−1^ that can be attributed to the intrinsic stretching of oxygen and iron at the tetrahedral sites, whereas a small signal at ca. 440 cm^−1^, outside of the instruments range, can be associated with the octahedral Fe-O stretch [35,36,37]. Small signals appearing at 1400 cm^−1^, 1623 cm^−1^, and around 2900 cm^−1^ can be due to hydrocarbon impurities and some moisture absorbed from ambient air. This strong band is slightly changed/split upon the modification with oleic acid and TWEEN 80 layer. Therefore, we can consider the absorption in the range of 1000 cm^−1^ to 1750 cm^−1^ as the diagnostic region of surface modification of SPIONs. These bands are visible in Figure 5 and Figure 6 [38]. Looking at the spectrum of pure TWEEN 80 in literature [39], we can find the bands at 1103 and 1738 cm^−1^ ascribed to the asymmetric C–O and C=O stretching vibrations of the surfactant. These bands can be found on the spectra of our conjugates. Pure CBD and CBG reveal a vibration coming from the C-H bond of the rings (cyclohexane or aromatic ring) at 3050–3070 cm^−1^, methyl and methylene at 2800–3000, around 1575 cm^−1^ was indicative of C=C stretching (phenyl ring), and C-O stretching vibrations were at ~1216 cm^−1^ [40]. We can see small signals coming from C-H bonds of aromatic rings of cannabinoids. The peak intensity is smaller for CBG than for CBD because CBG only has one aromatic ring. According to the spectra of pure epi, many more peaks are revealed. The band of 1380 cm^−1^ is assigned to the bending vibration of the alkyl group (CH_2_), while the presence of two peaks at 2840 cm^−1^ and 2910 cm^−1^ can be matched to the stretching vibration of the CH_2_. The bands that appear at 1276 cm^−1^ and 1210 cm^−1^ are derived from the vibration of enol (C–O). The band of 1632 cm^−1^ is characteristic of simultaneous stretching of C=C and C=O [41]. The spectra of conjugates showed a part of the characteristic peaks of CBD and CBG, because of the cannabinoids’ molecular dispersion or entrapment within organic bilayer onto nanoparticles. Several characteristic analyzed signals are marked in black frames on the spectra. Moreover, no additional peaks were observed in the spectra: SPION@OA_T80_CBD_epi and SPION@OA_T80_CBG_epi, indicating that the loading of cannabinoids or drug did not change the nature of nanocarriers. The bands coming from epirubicin overlap with those derived from cannabinoids due to similar regions in the structure of both molecules, such as the aromatic ring. Therefore, this study confirmed the presence of cannabinoids/epirubicin in conjugates.

### 3.4. Drug Content in Conjugates

The amount of cannabinoid incorporated in the conjugate was determined using differential scanning calorimetry. Since epirubicin is a redox-active molecule, to determine the quantity of epirubicin, cyclic voltammetry was used.

#### 3.4.1. Differential Scanning Calorimetry

**Figure 7:**
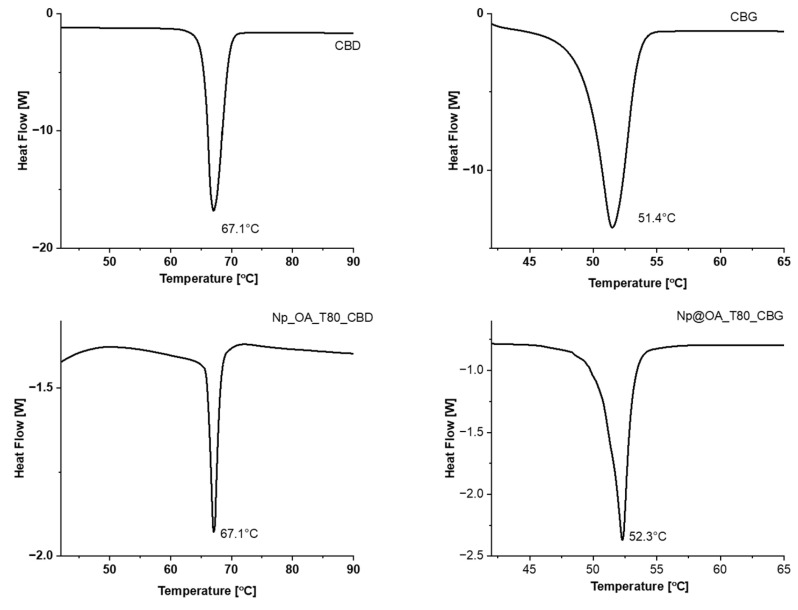
DSC thermograms for pure CBD (**top left**), CBG (**top right**), and the conjugates with CBD (**bottom left**) and CBG (**bottom right**).

Differential scanning calorimetry (DSC) thermograms for pure CBD and CBG show sharp endothermic peaks observed at 67.1 and 51.4 °C corresponding to the melting points of these compounds, as supported by literature (Figure 7). The DSC curves for conjugates also show sharp peaks at 67.1 °C and 52.3 (only insignificant shifts for CBG were observed) which indicate the presence of cannabinoids in their structure. By integrating the area under the peak, the values of enthalpy of melting, ΔH_melt_, were determined, which are 20.1 and 21.2 kJ/mol. These values are fairly similar to those reported in the literature, which are 20.3 kJ/mol for CBD [42,43]. Based on the enthalpy values of pure cannabinoids and the DSC thermograms for the conjugates, the CBD and CBG contents in the carriers were estimated at 9.2% and 8.9% per 1 g of nanoparticles [44].

#### 3.4.2. Cyclic Voltammetry

For electrochemical measurement, a three-electrode system was used, containing a GCE as the working electrode, Ag,AgCl|3 M KCl_aq_ as a reference electrode, and platinum wire as the counter electrode.

Initially, the GCE electrode was polished using a paste of 0.05–0.3 µm alumina. Then, the electrode was sonicated for 2 min in ethanol and deionized water to remove pollution from the surface. Then, 1 µL of SPION@OA_T80_CBD_epi or SPION@OA_T80_CBG_epi was dropped and dried at room temperature for 20 min.

The electrochemical measurement of epirubicin was carried out in the 10 mM phosphate buffer saline solution as the supporting electrolyte. The CV voltammograms were recorded over the potential from 0 to 1.0 V at a 100 mV/s scan rate.

**Figure 8:**
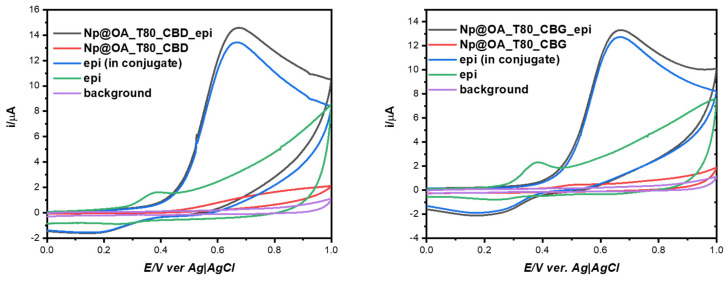
Cyclic voltammetry curves for SPION@OA_T80_CBD_epi (**left** panel, black curve) or SPION@OA_T80_CBG_epi (**right** panel, black curve). Blue curves show the background-subtracted CVs that were used to evaluate the amount of epirubicin contained in the conjugate. As background cyclic voltammograms we used conjugate deposited on the electrodes with no epirubicin (red curves). Purple voltammograms are for bare GCE electrode, whereas green curves are for epirubicin dissolved in the buffered supporting electrolyte.

The obtained results are shown in Figure 8. What can be inferred from this figure is that the anodic signal assigned to the −2e oxidation of epirubicin (Figure 8, black curves) is shifted toward more positive potential values than for the bare GCE electrode under the same conditions (green curves). We think that this result can be explained by the presence of conjugate CBD or CBG layer imposing larger resistance and consequently a higher energetic barrier to the electrochemical oxidation of epirubicin. In this figure blue curves are the result of subtracting the background currents (red curves, conjugates with CBD or CBG) from the black curves, allowing us to evaluate the amount of epirubicin incorporated in the conjugates and deposited on the GCE electrodes. This amount, in terms of mass percentage, was found to be 4.5% and 4.4% epirubicin per 1 g of SPION@OA_T80_CBD_epi, and SPION@OA_T80_CBG_epi, respectively [45].

### 3.5. Magnetic Hyperthermia Studies

The heating efficiency of conjugates was studied by magnetic hyperthermia, where the magnetic energy of SPIONs is converted into heat under the influence of the alternating magnetic field. The relaxation that generates the heat within the core is due to the Néel and Brown effects. Samples of 1 mL and a density of 20 mg/mL in water were placed in the copper coil, where they were thermostated. Experiments were performed with alternating magnetic field in three different frequencies (380, 487, and 633 kHz) and with three different magnetic field amplitudes (125, 188, and 251 G). Measurements were performed until reaching 55° C (328 K) or during 300 s. The measurements were followed by the evaluation of a specific absorption rate (SAR).

**Figure 9:**
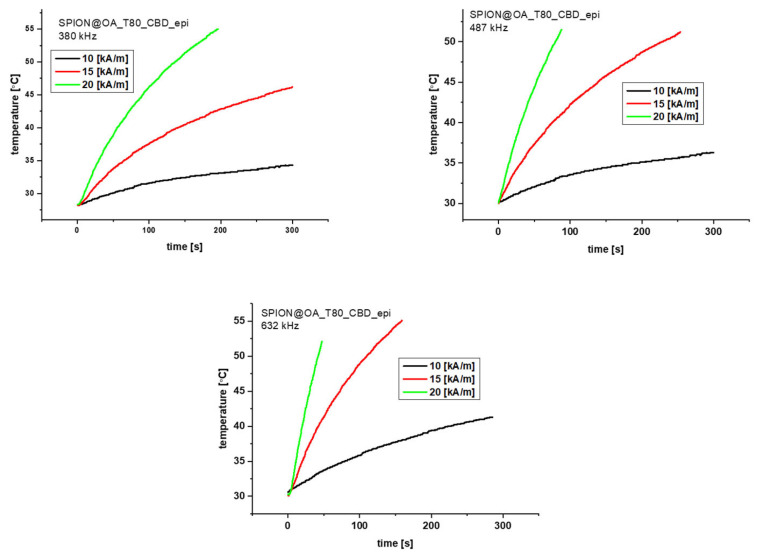
Magnetic Hyperthermia graphs for the CBD conjugate for 380, 487, and 633 kHz and three different fields 10 (black, 125 G), 15 (red, 188 G), and 20 (green, 251 G) kA/m.

**Figure 10:**
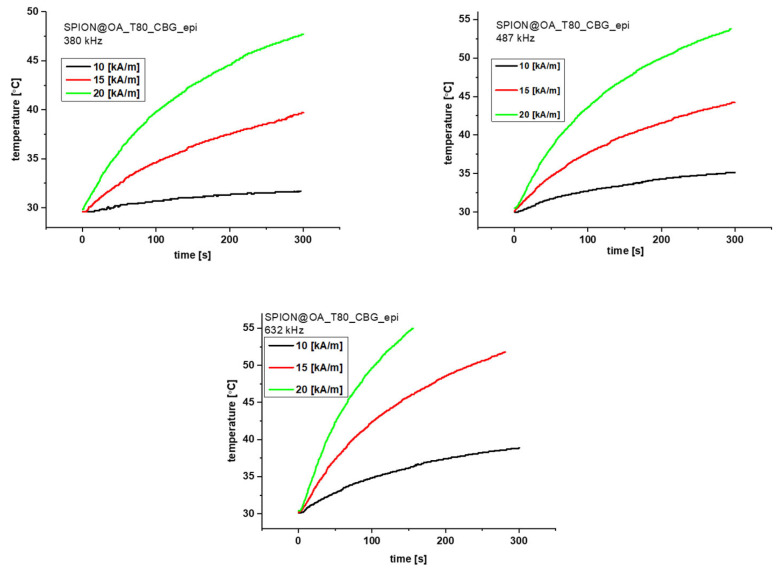
Magnetic Hyperthermia graphs for the CBG conjugate for 380, 487, and 633 kHz and three different fields 10 (black, 125 G), 15 (red, 188 G), and 20 (green, 251 G) kA/m.

Looking at Figure 9 and Figure 10, we can see that the graphs differ between the conjugates, depending on the cannabinoid present in the structure. Conjugates with CBG need more time to reach 50 °C compared to the CBD ones. Looking at 380 kHz frequency we can say that the magnetic field amplitude of 10 and 15 generate a rather low heat rate. In the span of 300 s, we barely see the increase in temperature. However, the result of 188 G for the CBD conjugate could be used on organs that are less resistant to high temperatures. The frequency of 487 kHz is the most optimal for both conjugates. The gradual increase in temperature can be seen for 188 G for CBD and 188 and 251 G for CBG. The temperature for 251 G for CBD rises too quickly so we cannot control it as much as for the other frequencies. Finally, looking at the last frequency of 633 kHz, we can see rapid temperature increases of 188 and 251 G for CBD and 251 G for CBG. The amplitude of 125 G for both conjugates can be used on organs less prone to heat. The only great result for this frequency is noticeable for the CBG carrier for the amplitude of 188 G. Based on the obtained graphs, we evaluated the SAR factor as follows:

Table 3 presents the obtained SAR values. The obtained SAR values are slightly lower than the typical SAR results shown in the literature for SPIONs. We need to remember, however, that our conjugates have a really large organic shell, responsible for 50% of the conjugate’s mass. Converting to the core alone, our results correspond to the literature values of 250–300 W/g [46].

### 3.6. Langmuir Technique

So far, many model systems have been developed to imitate the biomembrane environment. One of them is the Langmuir technique, the most powerful and simple method to obtain monolayers—one leaflet of a biological membrane. Monolayers are an excellent model for studying the processes occurring on the membrane surface, the interactions between its components, and the mechanism of drug-lipid interaction. The advantage of creating monolayers using the Langmuir method is the ability to control the degree of packing of molecules in the film, the composition of the subphase (pH, ionic strength), the temperature, the variety of techniques allowing the characterization of monolayers, and the simplicity of the model. The results of experiments using the Langmuir monolayer can be applied to lipid bilayers and provide essential information not only about the membrane components and their properties but also about the influence of biomolecules (e.g., drugs, carriers) on model cell membranes by verifying their affinity for the appropriate membrane components and determining their interactions.

Cancer cells have many adaptations that allow them to reduce the effectiveness of anticancer drugs. One of the most significant properties of cancer cells is the disturbed lipid metabolism and consequently the non-standard composition of the cell membrane compared to a healthy cell. There is no single, specific lipid profile characteristic of cancer cells that would clearly distinguish them from non-cancerous cells. Differences can be found in changes in the lipid content in the outer and inner layers of the cell membrane. In cancer cells, the lipid composition is reorganized, which results in the exposure of negatively charged phosphatidylserine (PS) groups. Increasing the amount of PS in the outer part of the membrane protects cancer cells from the action of Natural Killer (NK) cells and other cytotoxic immune cells. This ability causes cancer cells to not be recognized as foreign structures by the immune system. In the studies presented below, two lipids, DOPC and DOPS, were used to create monolayers modeling cell membranes. A Langmuir monolayer consisting only of the DOPC lipid will represent a model of a healthy cell membrane, while a mixture of DOPC and DOPS will be an equivalent of a cancer cell membrane. Thus, our studies focused on examining the changes in surface pressure caused by the addition of the conjugate or drug into the subphase as a function of time.

**Figure 11:**
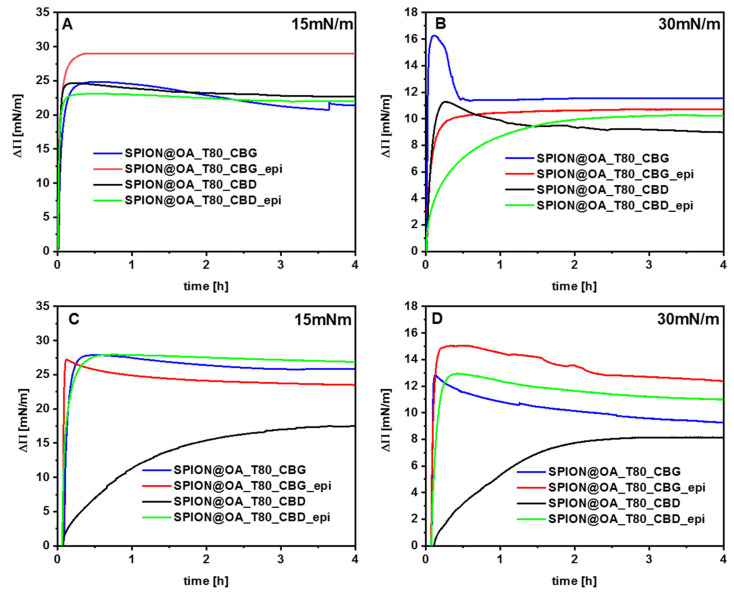
Changes of surface pressure in time for DOPC monolayer (**A**,**B**) and DOPC/DOPS monolayers (**C**,**D**) compressed to the surface pressure of 15 mN/m and 30 mN/m after the injection of drug or conjugates into the subphase (marked on figures).

In the first stage of experiments, the monolayers were compressed to the values of surface pressure Π = 15 mN/m (monolayer not well organized), and Π = 30 mN/m, the latter value as an equivalent of the organized cell membrane. After reaching the target pressure, the conjugate or drug solution was injected into the subphase (under the monolayer) and the surface pressure changes were recorded in time. The results are shown in Figure 11A–D. Please note that the initial changes observed in this Figure are due to the equilibration of the local concentration of SPIONs in the subphase after the injection. Therefore, below we will discuss the results recorded at least 1 h after the injection.

Epirubicin and conjugates easily penetrate the monolayers previously compressed to 15 mN/m, which is confirmed by a large increase in the surface pressure value, ΔΠ~20–25 mN/m (Figure 11A,C). In this situation, the membranes are not well-organized, and the lipids are not tightly packed, so the molecules from the subphase can be incorporated into the air–water system. When phospholipid molecules are more ordered on the water surface, i.e., the layer is compressed to a pressure of 30 mN/m, it is clearly visible that the components of the subphase penetrate its interior less intensively (Figure 11B,D ΔΠ~10–12 mN/m). However, an increase in surface pressure is still observed, which suggests the occurrence of interactions between the drug/conjugate and lipids forming the monolayer.

Comparing the curves for the CBG and CBD conjugates, it apparently seems that a stronger response (ΔΠ) is observed for the nanocarrier with cannabigerol. This can be explained by the difference in the structure of both cannabinoids. The molecule of CBD has two rings in the structure, which can cause steric hindrance and make it difficult for the compound to locate in the membrane.

For the model of the cancer cell membrane, the presence of the drug in the conjugate causes a greater increase in surface pressure compared to the nanocarrier with only cannabinoid. Of the two lipids forming this monolayer, DOPS has negatively charged headgroups that can electrostatically interact with positively charged epirubicin molecules. The polar lipid headgroups of DOPC are zwitterions, so electrostatic interactions between them and epirubicin are much more difficult than in the case of DOPS. The stronger interactions of the conjugate can be explained by the fact that the drug molecules first approach the polar region of the monolayer and electrostatic interactions are dominant. In this situation, even a high molecular order does not prevent anthracycline interactions, although it can be assumed that the drug molecules remain mainly in or under the region of the polar headgroup, where electrostatic attraction occurs.

The conducted studies pointed toward the possible synergism between cannabinoid and epirubicin; additionally, this effect is slightly stronger in the case of conjugates with CBG. Simultaneous application of natural compounds from *Cannabis sativa* L. and epirubicin may increase the effectiveness of chemotherapy due to the increased ability to penetrate the cell membrane. The possibility of synergism was followed by in vitro studies presented in the next section.

### 3.7. Cytotoxicity Results

**Figure 12:**
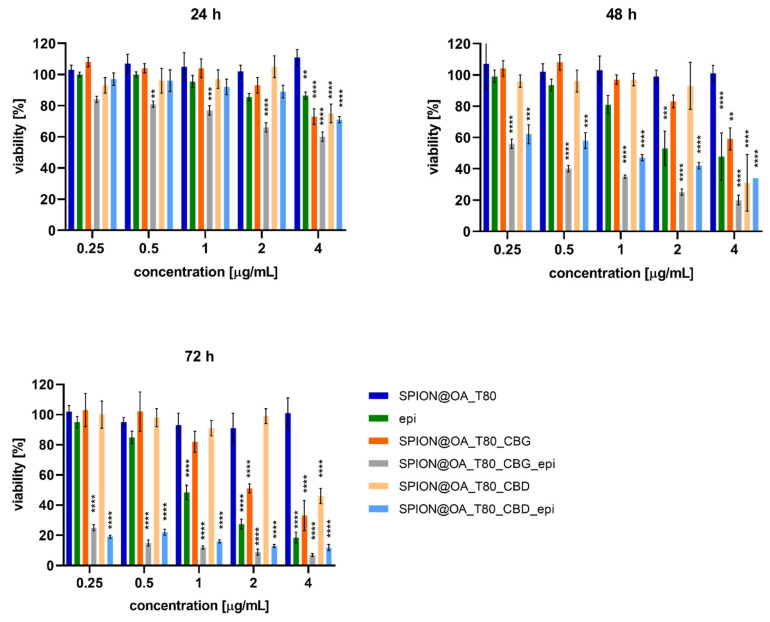
Viability of SKOV-3 cells treated with SPION@OA_T80, SPION@OA_T80_CBG, SPION@OA_T80_CBG_epi, SPION@OA_T80_CBD, SPION@OA_T80_CBD_epi and epi after 24, 48, and 72 h. For statistical analysis, cells treated with SPION@_T80 were used as a control. Results were considered statistically significant when *p* ≤ 0.01 (**), *p* ≤ 0.001 (***) and *p* ≤ 0.0001 (****). One may notice, in some cases, an apparent increase in absorbance readings, leading to viability values exceeding 100%. The MTS assay relies on mitochondrial reductase activity that correlates directly with the number of viable cells. However, natural variations in cellular metabolism (or even differences in cell seeding density during the experiments) may result in the observed values apparently exceeding 100%, without necessarily increasing cell number or viability.

In vitro cell studies using MTS reagent were performed to evaluate the cytotoxicity of SPIONs conjugated with CBD/CBG and epi. The nanoparticles coated with TWEEN 80 exhibited minimal cytotoxicity, which is expected, as TWEEN 80 is typically used as a surfactant and stabilizer without causing significant cellular damage. No cell death was observed even after 72 h of exposure. Notably, cytotoxicity increased in a time- and concentration-dependent manner for all tested formulations, including conjugates with cannabinoids, epirubicin, and their combinations. Cannabinoids alone, specifically CBD and CBG, exhibited cytotoxicity, with approximately 60–70% of cancer cells killed at the highest concentrations tested. This suggests that cannabinoids have some inherent anticancer properties, though they are not sufficient to fully eradicate the cancer cells. Approximately 30–40% of the treated cells survived, indicating that the treatment may only cause transient damage. Epirubicin, a potent chemotherapy drug, showed strong therapeutic effects, killing approximately 80% of cancer cells at the highest concentrations. However, the remaining 20% of cells survived, which may be attributed to variations in drug uptake, resistance mechanisms, or limited penetration of the drug within the cell culture. The combination of cannabinoids (CBD or CBG) with epirubicin demonstrated a significant enhancement in cytotoxicity. The treatment resulted in less than 10% cell viability at the highest concentrations after 72 h. The results of the cytotoxicity studies are shown in Figure 12. This reduction in cell survival indicates a pronounced synergistic effect between the cannabinoids and epirubicin, where cannabinoids likely enhance the drug’s efficacy in killing cancer cells. This synergy was further confirmed through statistical analysis.

Using the CompuSyn program, synergy factors (CI) were determined empirically for 50% survival after 72 h along with the dose reduction index (DRI), for the same survival threshold.

The synergy factors for the CBD and CBG conjugates were 0.00099 and 0.023, respectively, indicating very strong synergistic interactions between cannabinoids and epirubicin. These low values confirm that the combination enhances anticancer activity. The DRI values further support this conclusion, demonstrating the extent to which drug doses can be reduced while maintaining the same therapeutic effect. For the highest tested concentration, the dose of CBD could be reduced by 366%, and the dose of epirubicin by 108%. Similarly, for the conjugate with CBG, the cannabinoid dose could be reduced by 144%, and the anthracycline concentration by 154%. These results align with the observed therapeutic effects of the drugs when administered separately versus in combination. The enhanced cytotoxicity observed in the combination treatment highlights the importance of this approach, where cannabinoids not only improve the efficacy of epirubicin but also potentially reduce the chances of tumor recurrence by targeting multiple aspects of cancer cell biology.

## 4. Conclusions

We present a nanoparticulate carrier system based on SPIONs to incorporate hydrophobic cannabinoids and hydrophilic anthracycline drug. The obtained conjugates have a size of about 100 nm. The cores of the carrier were SPIONs, which were subjected to magnetic hyperthermia at three different field frequencies and three different magnetic field amplitudes. Studies conducted with the Langmuir monolayers indicate a strong interaction between the cannabinoids and lipid monolayer, pointing toward possible facilitation of the conjugate’s penetration through the cell membrane. The drug enhances this effect, confirming the synergism between the active compounds of *Cannabis sativa* L. and epirubicin. Lastly, the cytotoxicity tests were conducted against the SKOV-3 cell line in vitro. We observed a high synergistic effect between cannabinoids and epirubicin, which we confirmed with a statistical analysis. We think that the observed cytotoxic effect may be related also to the influence of cannabinoids on the structure of lipid membranes. Due to their structural similarity to cholesterol, cannabinoids can penetrate the structure of biological membranes and change the fluidity of lipids, which affects the properties of the membrane. Therefore, it is possible that the cannabinoid from the conjugate acts as a pre-interacting factor with the biological membrane by influencing its fluidity and properties. This step contributes to the destabilization of the membrane and facilitates the penetration of active compounds into the cell. We are also inclined to think that the usage of magnetic hyperthermia can not only stimulate cell death, but also, due to the local temperature rise, enhance the release of cannabinoids and epirubicin from the conjugates. However, the results presented in this work focused mainly on the chemical aspects of carriers’ development and characterization. Much more complex studies should be performed, including ultrastructural TEM analysis, detailed internalization studies, and apoptosis assays with cell cycle analysis. These studies are ongoing in our group.

## Figures and Tables

**Table 1:** The table consists of the average diameter and Zeta potential of all obtained conjugates.

Carrier	Average Diameter [nm]	Average Zeta Potential [mV]
SPION@OA_T80	65 (±3)	0.7 (±0.2)
SPION@OA_T80_CBD	90 (±2)	−12 (±4)
SPION@OA_T80_CBG	87 (±4)	−13 (±5)
SPION@OA_T80_CBD_epi	98 (±5)	−2.7 (±1.2)
SPION@OA_T80_CBG_epi	102 (±3)	−3.9 (±1)

**Table 2:** Elemental composition of samples.

Sample	%C	%O	%Si	%Fe	Cl
SPION@OA	90.7	8.2	1	0.1	
SPION@OA_T80	74.7	21.3	1.2	2.23	0.6
SPION@OA_T80_CBD	86	13.4		0.6	
SPION@OA_T80_CBD_epi	85	14.4		0.6	

**Table 3:** Specific absorption rate (SAR) values for two conjugates.

**SPION@OA_T80_CBD_epi**	10 [kA/m]	15 [kA/m]	20 [kA/m]
380 [kHz]	27 [W/g]	61 [W/g]	91 [W/g]
487 [kHz]	36 [W/g]	77 [W/g]	132 [W/g]
633 [kHz]	60 [W/g]	82 [W/g]	152 [W/g]
**SPION@OA_T80_CBG_epi**	10 [kA/m]	15 [kA/m]	20 [kA/m]
380 [kHz]	31 [W/g]	60 [W/g]	96 [W/g]
487 [kHz]	37 [W/g]	78 [W/g]	143 [W/g]
633 [kHz]	62 [W/g]	83 [W/g]	160 [W/g]

## Data Availability

Data available within the article.

## Supplementary Materials

The following supporting information can be downloaded at: https://www.mdpi.com/article/10.3390/biom15020230/s1, Figure S1: Magnetization measurement for SPION@OA_T80_CBD_epi (upper panel) and SPION@OA_T80_CBG_epi (lower panel). Insets—the magnetization loop in the range +/− 100 Oe.
