# Supplementary material for: Tailored Iron Oxide Nanoparticles as Potential Cannabinoid Carriers for Anti-Cancer Treatment
Source: Biomolecules. 2025 Feb 5;15(2):230. doi: 10.3390/biom15020230 (PMC11853022; doi:10.3390/biom15020230)
Supplement: Supplementary file 1 [file biomolecules-15-00230-s001.zip › biomolecules-3376944-supplementary.pdf]

Supplementary Materials

S1. Magnetic Properties of Conjugates

To investigate the magnetic properties and evaluate further possibilities of using the conjugates in magnetic hyperthermia, magnetization measurements were performed. Figure S1 shows the recorded magnetization hysteresis curves for both conjugates. The obtained results prove that the nanoparticles exhibit good magnetic properties. It turns out that a magnetic field of about 2000 Oe is sufficient to saturate the samples, and the saturation magnetization values Ms (caculated per core of conjugate) are 59 and 61 emu/g for SPION@OA_T80_CBD_epi and SPION@OA_T80_CBG_epi, respectively. Comparing the black and red curves for both conjugates (Figure S1; upper and lower panel), you can see that the presence of organic shell causes some decrease in magnetic properties. This behavior is typical for a magnetic core covered with a diamagnetic organic layer. The presented values of Ms qualify the obtained conjugates for applications in magnetic hyperthermia, which is discussed in section 3.5 (in the main text). The insets in figure S1 show the magnetization of conjugates at a very low external magnetic field (± 100 Oe). As can be seen in this range both samples exhibited negligible coercivity and remanence which can relate to the superparamagnetic features that can be used in magnetic hyperthermia studies. Additionally, the low values of magnetic remanence hinder the aggregation and precipitation of nanoparticles from the solution.

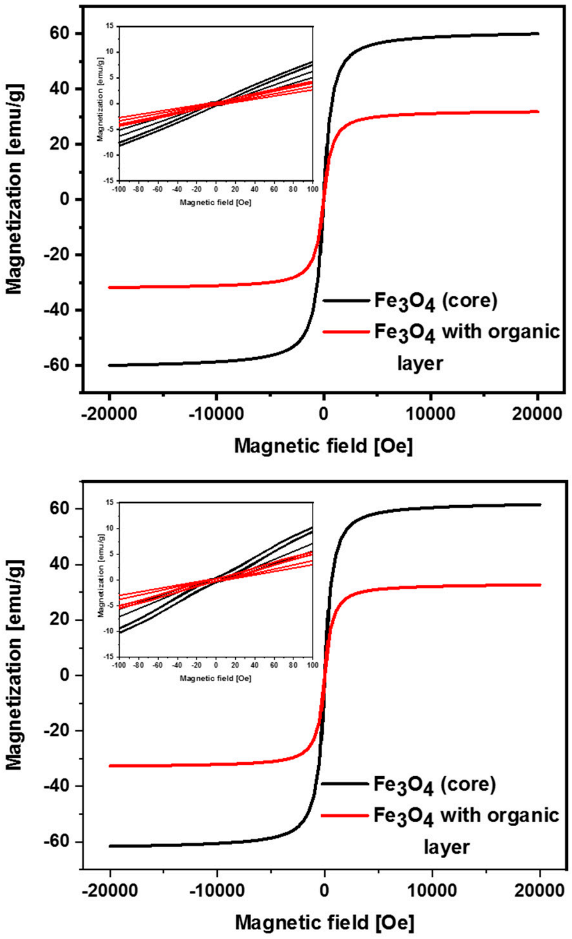

Figure S1. Magnetization measurement for SPION@OA_T80_CBD_epi (**upper panel**) and SPION@OA_T80_CBG_epi (**lower panel**). Insets—the magnetization loop in the range +/− 100 Oe.
